# Menorrhagia as main presentation sign of severe hypothyroidism in a pediatric patient: a case report

**DOI:** 10.1186/s13052-022-01363-x

**Published:** 2022-09-11

**Authors:** Arianna Barbero, Manuela Pagano, Gerdi Tuli, Raffaele Buganza, Luisa de Sanctis, Claudia Bondone

**Affiliations:** 1 Department of Pediatric and Public Health Sciences, Postgraduate School of Pediatrics, Regina Margherita Children’s Hospital, University of Turin, Turin, Italy; 2 Department of Pediatric Emergency, Regina Margherita Children’s Hospital, AOU Città Della Salute E Della Scienza, Turin, Italy; 3 Department of Pediatric Endocrinology, Regina Margherita Children’s Hospital, AOU Città Della Salute E Della Scienza, Turin, Italy

**Keywords:** Menorrhagia, Hypothyroidism, Bleeding risk, Coagulation

## Abstract

**Background:**

The relative high frequency of menstrual irregularities in the first two–three years after menarche may lead to the risk of underestimation of associated pathological conditions, which are always to be accurately researched with careful examination and anamnesis. The association between menstrual irregularities and hypothyroidism is described in literature but the available data are scarce and mainly based on adult case series. It is described that low plasma levels of thyroid hormone can shift the hemostatic system towards a hypocoagulable and hyperfibrinolytic state and seem to lead to an increased bleeding risk.

**Case presentation:**

This case report describes the case of a thirteen years old girl who presented to our Emergency Department complaining of menorrhagia for the last fifteen days, leading to severe anemia. The objective examination revealed clinical signs of hypothyroidism and a severe short stature, lower than mid-parental height, with stunting of growth and a significant bone age delay. Blood exams and thyroid ultrasound were consistent with the diagnosis of severe hypothyroidism in autoimmune thyroiditis with acquired von Willebrand syndrome, growth hormone deficiency. Magnetic resonance showed pituitary functional hyperplasia.

The substitutive therapy with levothyroxine led to the resolution of heavy bleeding after five days and following normalization of coagulative parameters and pituitary hyperplasia.

**Conclusions:**

Hypothyroidism usually presents with unspecific symptoms, with consequent risk of diagnostic delay. It can influence the coagulation system and it seems to be associated to increased risk of menstrual irregularities.

We underline the importance of a regular follow up of the pubertal development, including height measurements, thyroid palpation and menstrual anamnesis to intercept red flags findings for hypothyroidism.

## Background

Autoimmune hypothyroidism (Hashimoto thyroiditis) is the most common cause of acquired hypothyroidism in children, adolescents and adults [1], with an estimated prevalence of 1–2% in pediatric age [2].

Hypothyroidism usually presents with unspecific symptoms, as fatigue, weight gain, growth retardation, cold intolerance, constipation [2]. The goiter is the most common physical sign, other examination findings include bradycardia, delayed reflexes and myxedema [1].

Furthermore, thyroid hormone affects different cardiovascular parameters, for example hypothyroidism is associated with an unfavorable lipid profile [3]. Although less known, it also influences the coagulation system: low plasma levels of thyroid hormone can shift the hemostatic system towards a hypocoagulable and hyperfibrinolytic state and seem to lead to an increased bleeding risk, which could be relevant in particular in patients undergoing invasive procedures [4]. Hypothyroidism seems also to be associated to increased risk of abnormal vaginal bleeding, but the available data are scarce [4] and mainly based on adult case series. In women with hypothyroidism, different changes in menstrual cycle are reported [5]: the most common form is oligomenorrhea [6] and other manifestations include heavy bleeding, amenorrhea, breakthrough bleeding [7].

Excessive or unpatterned uterine bleeding is normal in adolescents without thyroid disease in the first years after menarche [8]. This can lead to underestimation of the possible pathological causes at the basis of menstrual irregularities, in particular in the absence of other associated symptoms, which must always be sought carefully.

We present a case of a pediatric patient with menorrhagia, which led to severe anemia, due to hypothyroidism in autoimmune thyroiditis with alteration of the coagulation system.

## Case presentation

A Salvadoran thirteen years old girl presented to our Emergency Department complaining of menorrhagia for the last fifteen days. She had menarche one year and seven months before, followed by absence of menstruation until the previous month, when she had a menstruation normal in duration and flow. She did not report asthenia nor other symptoms. Family history was negative for coagulopathy, the mother had hypothyroidism in pregnancy and the father had hyperthyroidism.

The objective examination revealed xerotic and desquamating skin, thinning hair and acanthosis nigricans on neck, armpits and ankles. She presented a mildly enlarged thyroid gland at palpation, also visible at extended head, heart rate was 54 beats per minute, nothing relevant was noted at abdominal and pulmonary examination.

She was 133.8 cm tall (-3.53 standard deviation scores, SDS [9]), her mid-parental height was 155 cm (- 1.25 SDS [9]), weight was 44 kg (-0.32 SDS [9]) and Body Mass Index, BMI 24.6 (1.37 SDS [9]). The mother referred stunting of growth in the last two years but no previous height measurements were available.

Abdominal ultrasound showed a vaginal anechoic formation (63 × 61x34 mm) with a non-vascularized hyperechoic structure inside (43 × 36x25 mm), compatible with blood collection with a clot inside, which was spontaneously expelled few hours later, and a right ovarian cyst (40 × 28 mm).

The first blood tests revealed decreased hemoglobin (8 g/dl, N.V. 11.3–14.5 g/dl) and red blood cells (2.63 10^12/L, N.V. 4–5.1 10^12/L), normal prothrombin (PT) ratio, slightly increased activated partial thromboplastin time (aPTT) ratio (1.27, N.V. 0.8–1.18) and mildly decreased levels of fibrinogen (169 mg/dL, N.V. 200–400 mg/dL), von Willebrand Ristocetin Cofactor assay (vWF: Rco) (33%, N.V. 60–200) and von Willebrand factor (VWF) (39%, N.V. 66–176)*.*

Thyroid ultrasound was performed for the suspect goiter, confirming an enlarged thyroid (lobes measuring 17 mm in anteroposterior diameter, isthmus 5.5 mm), with non-homogeneous hypoechogenic structure and hyperechoic linear bands, two hyperechogenic nodules (3 mm) in the right lobe, an increased vascularization and some reactive perithyroid lymph nodes, the major measuring 18 mm in his long axis.

Blood exams to evaluate thyroid function showed an increased thyrotropin releasing hormone (TSH) level (> 100 mU/ml, N.V. 0.660–5.060 mIU/L), with extremely low free thyroxin (fT4) in serum (< 1.5 ng/L, N.V. 7.4–13.5 ng/L), high titer of anti-thyroid peroxidase antibodies (TPOAb) (> 1000 kUI/L, N.V. < 10 kUI/L) and antithyroglobulin antibodies (TGAb) (> 1000 kUI/L, N.V. < 10 kUI/L) and negative TSH receptor autoantibodies (TRAb).

The patient was consequently diagnosed with autoimmune hypothyroidism and the substitutive therapy with levothyroxine was started at 50 mcg/die, then increased to 100 mcg/die after two weeks.

Autoimmune conditions potentially related to Hashimoto thyroiditis (such as type I diabetes, coeliac disease, autoimmune gastritis, hypoparathyroidism and Addison disease) were excluded.

In the second day of hospitalization, she required a red blood cells transfusion for worsening of anemia (hemoglobin 6,3 g/dl). Heavy vaginal bleeding stopped in the fifth day of hospitalization, with subsequent spotting until the following menstruation on the fifteenth day. This menstruation, as the following ones, was still abundant in flow, but lasted for five days.

Cerebral magnetic resonance (MR) was performed to evaluate the possible pituitary functional hypertrophy, with the finding of an enlarged adenohypophysis (16 mm in height) in contact with optic chiasm. Left hand X-Ray showed a bone age of 9.6 years according to TW2 RUS method [10].

The patient reached fT4 normalization after four weeks and TSH normalization after seven weeks of therapy. One month after the beginning of levothyroxine, coagulative parameters were re-dosed with a normalization of aPTT ratio (1.03), fibrinogen (322 mg/dL), vWF: Rco (65%) and VWF levels (83%).

Growth hormone (GH) release after arginine and glucagon tests was dosed one and two months after the beginning of therapy and resulted insufficient (respectively GH 0.61 UI/l, N.V. < 8 UI/l and GH 1.43 mcg/l, N.V. < 8 UI/l), with the diagnosis of GH deficiency. The patient was consequently prescribed with substitutive growth hormone therapy after the second MR, showing resolution of the pituitary hyperplasia after three months of therapy.

## Discussion

Our patient’s main presenting symptom was menorrhagia, however menstrual irregularities are very frequent in the first two–three years after menarche due to the immaturity of the hypothalamic-pituitary-ovarian axis [11]. The relative high frequency of those irregularities may lead to the risk of underestimation of associated pathological conditions, which are always to be accurately researched with careful examination and anamnesis. In our case the patient presented short stature, significantly lower to the predicted height (considering also the pubertal stages with menarche 1.7 years before), and other signs of hypothyroidism, as bradycardia despite the anemia, mildly enlarged thyroid, dry skin, thinning hair.

The association between menstrual irregularities and hypothyroidism is described in literature: the hypothalamic-pituitary-thyroid axis and the hypothalamic-pituitary–gonadal axis work together, and any dysfunction in the thyroid can affect the serum sex steroid levels, sex hormone-binding globulin (SHBG), gonadotropin-releasing hormone (GnRH) and prolactin [7]. The prevalence of menstrual irregularities in hypothyroidism has been investigated in adult series, but not in a pediatric setting. Furthermore, there seems to be conflicting evidence.

Joshi et al. found that 68% of hypothyroid women had menstrual irregularities, compared with only 12% in controls [12]. Krassas et al. reported that among 171 hypothyroid women, 23% presented irregular cycles (compared with only 8% in controls); of those, 17 had oligomenorrhea, 6 hypomenorrhea, 6 amenorrhea, and 12 hypermenorrhea/menorrhagia [13]. Among the menstrual irregularities described in literature the most common form is oligomenorrhea [6] and there is an increase in the occurrence of menstrual irregularities with increase in severity of hypothyroidism [14]. On the other hand, Kakuno et al. reported that the incidence of menstrual disturbances was similar among 586 women with hyperthyroidism (18.3%), 111 women with hypothyroidism (15.3%) and 105 healthy controls (23.8%) [15].

In our patient the main presentation sign was menorrhagia with heavy bleeding. Hypothyroidism seems to affect the coagulative cascade in different ways, shifting the hemostatic system towards a hypocoagulable and hyperfibrinolytic state [4]. Acquired von Willebrand syndrome (aVWS) is the most frequent coagulation disorder clinically observed in overt hypothyroidism [16]. The pathogenesis of hypothyroidism-associated aVWS is still unclear. A decrease in von Willebrand factor (VWF) protein synthesis or a decreased response to adrenergic stimulation (otherwise enhancing the VWF release from endothelial cells) due to the hormone deficiency are the most plausible mechanisms involved, as also supported by the finding of a reversal of the hypothyroidism-associated aVWS following thyroid hormone replacement [17].

In our patient VWF was assessed quantitatively and qualitatively, with an initial finding of decreased level and activity of the factor, which normalized at the control after one month of substitutive therapy.

Other alterations seem to be involved in this condition of hypocoagulability, like a significant reduction in coagulation factor VIII (FVIII), factor IX (FIX), and factor XI (FXI) levels [18] and qualitative platelet abnormalities [19].

Chadarevian et al. studied the fibrinolytic system in hypothyroid patients and documented a different fibrinolytic pattern according to the severity of hypothyroidism: an increased fibrinolytic activity (i.e., low levels of a2- antiplasmin, tissue plasminogen activator (tPA) and plasminogen activator inhibitor (PAI-1), and high Ddimer levels) was observed in overt hypothyroidism, whereas a decreased fibrinolytic activity (high levels of a2-antiplasmin, tPA and PAI-1, and low D-dimer levels) was found in subclinical hypothyroidism [20].

As regards the other findings in our case, the short stature with stunting of growth should have been investigated before with exams including thyroid function. Growth retardation is a common sign in hypothyroidism, due to the great importance of thyroid hormones in growth process.

Free triiodothyronine (fT3) is a primary determinant of normal post-natal somatic growth and skeletal development, and an important regulator of bone and mineral metabolism in the adult [21]. Before puberty, thyroid hormone may be the major prerequisite for normal maturation of bone with untreated childhood hypothyroidism resulting in profound growth retardation and delayed skeletal maturation [22]. This failure of growth is probably caused by a decrease of the direct effects of thyroid hormones on skeletal growth and by a secondary reduction in GH secretion and concentration of insulin-like growth factor-1 (IGF-1) [23].

In our patient, the short stature, significantly lower than mid-parental height, and the apparent stunting of growth were associated with growth hormone deficiency.

Prolonged severe hypothyroidism led also to pituitary functional hyperplasia. It is caused by high thyrotropin-releasing hormone (TRH) levels, that stimulate pituitary thyrotrope cells, leading to the enlargement of the pituitary gland [24]. According to this mechanism, with the normalization of the thyroid function we also saw the complete regression of the pituitary hyperplasia after three months.

## Conclusions

Menstrual irregularities, despite being very frequent in the first two years after menarche, can be a hallmark of underlying pathological conditions, whose signs and symptoms should be carefully researched, as short stature or stunting of growth and unexplained bradycardia for hypothyroidism, which can lead to a state of hypocoagulability.

We underline the importance of a regular follow up of the pubertal development, including height measurements, thyroid palpation, general examination and menstrual anamnesis to intercept red flags findings for thyroid disturbances.

## Data Availability

Data sharing not applicable to this article as no datasets were generated or analyzed during the current study.

## Abbreviations

APTT: Activated partial thromboplastin time
aVWS: Acquired von Willebrand Syndrome
BMI: Body Mass Index
FVIII: Factor VIII
FIX: Factor IX
FXI: Factor XI
fT3: Free triiodothyronine
fT4: Free thyroxin
GnRH: Gonadotropin-releasing hormone
GH: Growth hormone
IGF-1: Insulin-like growth factor-1
MR: Magnetic resonance
PAI-1: Plasminogen activator inhibitor
PT: Prothrombin
SDS: Standard deviation scores
SHBG: Sex hormone-binding globulin
TGAb: Antithyroglobulin antibodies
tPA: Tissue plasminogen activator
TPOAb: Anti-thyroid peroxidase antibodies
TRAb: TSH receptor autoantibodies
TRH: Thyrotropin-releasing hormone
TSH: Thyrotropin releasing hormone
VWF: Von Willebrand factor
vWF: Rco: Willebrand Ristocetin Cofactor assay
